# OPTIGOV - A new methodology for evaluating Clinical Governance implementation by health providers

**DOI:** 10.1186/1472-6963-10-174

**Published:** 2010-06-21

**Authors:** Maria Lucia Specchia, Giuseppe La Torre, Roberta Siliquini, Silvio Capizzi, Luca Valerio, Pierangela Nardella, Alessandro Campana, Walter Ricciardi

**Affiliations:** 1 Clinical Governance Unit, Institute of Hygiene of the Catholic University of the Sacred Heart, Largo Francesco Vito 1, 00168 Rome, Italy; 2 Clinical Medicine and Public Health Unit, Sapienza University, Viale Regina Elena, Rome, Italy; 3 Public Health and Microbiology Department, University of Turin, Via Santena, Turin, Italy; 4 Eurogroup Consulting, via del Porto Fluviale, Rome, Italy

## Abstract

**Background:**

The aim of Clinical Governance (CG) is to the pursuit of quality in health care through the integration of all the activities impacting on the patient into a single strategy.

OPTIGOV (Optimizing Health Care Governance) is a methodology for the assessment of the level of implementation of CG within healthcare organizations. The aim of this paper is to explain the process underlying the development of OPTIGOV, and describe its characteristics and steps.

**Methods:**

OPTIGOV was developed in 2006 by the Institute of Hygiene of the Catholic University of the Sacred Heart and Eurogroup Consulting Alliance. The main steps of the process were: choice of areas for analysis and questionnaire development, based on a review of scientific literature; assignment of scores and weights to individual questions and areas; implementation of a software interfaceable with Microsoft Office.

**Results:**

OPTIGOV consists of: a) a hospital audit with a structured approach; b) development of an improvement operational plan. A questionnaire divided into 13 areas of analysis is used. For each area there is a form with a variable number of questions and "closed" answers. A score is assigned to each answer, area of analysis, healthcare department and unit. The single scores can be gathered for the organization as a whole.

The software application allows for collation of data, calculation of scores and development of benchmarks to allow comparisons between healthcare organizations. Implementation consists of three stages: the preparation phase includes a kick off meeting, selection of interviewees and development of a survey plan. The registration phase includes hospital audits, reviewing of hospital documentation, data collection and score processing. Lastly, results are processed, inserted into a final report, and discussed in a meeting with the Hospital Board and in a final workshop.

**Conclusions:**

The OPTIGOV methodology for the evaluation of CG implementation was developed with an evidence-based approach. The ongoing adoption of OPTIGOV in several projects will put to the test its potential to realistically represent the organization status, pinpoint criticalities and transferable best practices, provide a plan for improvement, and contribute to triggering changes and pursuit of quality in health care.

## Background

The ever-increasing public concern over patient safety and quality in health care was the key driver of the development of Clinical Governance [1-3].

In 1997, the UK Department of Health published the White Paper "The New NHS: modern, dependable" [4], which introduced the concept of CG as a method of accounting for clinical quality in health care. However, CG really came to prominence in 1998 when Scally and Donaldson, in the British Medical Journal, appraised CG as the key drive towards quality improvement in the National Health Service (NHS). In this paper CG has been defined as "a system through which healthcare organizations are accountable for continuously improving the quality of their services and safeguarding high standards of care by creating an environment in which excellence in clinical care will flourish" [5].

The paper highlighted four components of quality, initially identified by the World Health Organization:

- Professional performance (technical quality)

- Resource use (efficiency)

- Risk management (risk of injury or illness associated with the service provided)

- Patient satisfaction with the service provided.

These four components formed the basis of CG in the Scottish and English Health Service [6].

Since the publication of the White Paper, CG profile and emphasis on its application have been growing steadily and a number of international healthcare systems have embraced the principles of CG, so that these are now of value worldwide in terms of quality, effectiveness and accountability [7-10]. In Italy, the principles of CG were initially mentioned in the January 14^th^, 1997 Republic President Decree (RPD), with particular reference to:

- the need for quality to be guaranteed by health care providers and to be monitored through specific indicators;

- the opportunity for the Patient/Citizen to freely choose among different health care organizations all meeting the same quality standards.

To this purpose, the 1997 RPD established the "minimum structural, technological and organizational accreditation criteria" (i.e. minimum number of rooms, minimum size of rooms, emergency lighting, medical gas plant among structural criteria; minimum availability of equipment for diagnostic and therapeutic services among technological criteria; number of doctors, nurses and technicians among organizational criteria). They are specific requirements that health care organizations have to meet in order to be permitted to provide health care in Italy, and which are verified by Regional authorities in the setting of a formal process. CG has been subsequently defined in the 1999-2001 Regional Healthcare Plan of Emilia Romagna, the first Italian Region to implement CG elements, as: "the heart of health organizations". The plan asserts the need to use suitable tools to avoid risks, quickly pinpoint adverse events, learn from mistakes, encourage good clinical practice and continuously improve it [11].

In a field characterized by plenty of tools for measuring and improving quality, CG aims to reveal that quality can be improved only by a general vision of organization, whose keys are the functional relationships among the different parts of the system [12,13]. CG is directed towards the integration of all the activities impacting on the patient into a single strategy, with different key components: research and development; education; continuous training and professional development; evidence based practice; clinical audits; clinical practice variability reduction; clinical leadership; team working and partnership promotion; performance measurement and appraisal; clinical risk management; patients and health care professionals involvement [14,15].

With the aim of assessing the implementation level of CG prerequisites and areas within an healthcare organization, trigger changes in clinical practice and promote a quality-oriented culture, a methodology called "OPTIGOV" (Optimizing Health Care Governance) was developed between March and December 2006 in Rome by the Department of Public Health of the Catholic University of the Sacred Heart, with the technical support of Eurogroup Consulting Alliance. OPTIGOV is addressed to health care professionals, both those with managerial roles (managing director, administration director and managers) and those with an assisting role with the patient within the clinical process.

The aim of this paper is to explain the process underlying the development of OPTIGOV, and describe in detail its constitutive elements, characteristics and steps.

## Methods

### Choice of analysis areas and questionnaire development

In January 2006, a multidisciplinary team was established consisting of 6 CG experts with a medical background (physicians expert in public health), 1 expert in governance and business management with a non medical background and 1 software developer. The team performed a systematic review of scientific literature consistently with the QUOROM statement (the PRISMA statement is the updated version currently available) [16,17]. PubMed and Embase databases and the key words "Clinical Governance"; "Quality of Health Care"; "Health Services Evaluation" were used. The time limits of the research ranged from January 1^st ^1996 to October 15^th ^2006. In PubMed 705 citations, in Embase 571 citations were found. A total number of 321 articles were selected based on title and abstract reading: 116 were full text articles, 69 of which were used by the team to develop the OPTIGOV methodology, based on the following internal inclusion criteria:

- CG definition;

- CG prerequisites and areas definition and description;

- description of best practices of implementation of CG areas;

- attempts to measure the level of application of CG practices in single health care structures;

- health care and services quality indicators.

The review was performed by 4 of the 6 experts of CG with a medical background (junior researchers) and any disputes were resolved trough the consultation of the other 2 experts (senior researchers) [16,17].

The most explanatory definitions of CG were looked up, beginning with the basic reference sources of the discipline that put into motion discussion on CG, the subsequent observations by the same Authors, as well as works on Evidence-Based Medicine [5,18-23]. Areas to which the perspective of CG has been applied within health care organizations since the introduction of the notion were taken into account. In each area, best practices were identified based on evidence on their efficacy, effectiveness and impact on quality improvement. Each classical area of CG was broken down into a series of constituent aspects (subareas) and each subarea was associated with one or more best practices. Previous attempts to measure the level of application of CG practices in single health care structures and indicators suitable for the measurement of the degree of application of the best practices so far identified were taken into account [24-26].

The team devised a series of questions capable of assessing the selected practices. Each area was associated with a form consisting of primary questions ("mother questions"), further developed by a series of secondary questions ("child questions"). The secondary questions have the purpose of specifically evaluating the single aspects of each global area. In choosing and phrasing the questions, a series of practical needs were taken into account. To safeguard objectivity and reproducibility, multiple choice questions were chosen. To ensure ease of application, questions were kept as short as possible, and their number as limited as possible, without compromising a complete coverage of all areas of CG.

Thanks to a pilot study carried out within a Scientific Research and Care Institute in Rome, the team validated the questionnaire by checking the questions were easy to understand and not liable to misinterpretation for either the interviewer or the interviewee, and by identifying the professional position that would have best answered the questions for each area.

### Scoring system

Each question was assigned a score, so that all the answers totalled up to a maximum global score of 100 for each area of analysis. The global score for CG was determined to be obtained by assigning each area a weight of 1/13.

#### Level of interest of the questions

The level of interest of the questions was chosen by some authors (WR and GLT) according to the level of recommendation and criteria suggested by the scientific literature.

- Every question considered to have the lowest level of interest was designated with *C*.

- Every question considered to have an intermediate level of interest was designated with *B*, equal to 2*C*s.

- Every question considered to have the highest level of interest was designated with *A*, equal to 2*B*s and to 4*C*s.

#### Weighing system

- All *A*, *B *and *C *questions were counted.

- The number of *C *questions represented the base.

- Since *B *= 2*C*s, the number of *B *questions was multiplied by 2.

- Since *A *= 4*C*s, the number of *A *questions was multiplied by 4.

- The maximum weight of all the answers of the form (100) was divided by the total of the result from *A*, *B *and *C *and the weight of the answer to *C *questions was obtained (if the form presented only questions *A *and *B*, the maximum weight of all the answers of the form (100) was divided by the result from *A *and *B *to obtain the weight of the answer to *B *questions).

- The weight of the answer to *C *questions was multiplied by 2 (*B *= 2*C*s) to obtain the weight of the answer to *B *questions.

- The weight of answer to *C *questions was multiplied by 4 (*A *= 4*C*s) to obtain the weight of the answer to *A *questions.

#### Assignment of weights to the forms (areas)

Different types of health care institutions have different callings and features, and cannot be expected to devote the same degree of effort to the same areas. Accordingly, a different weighing system for questionnaire forms (areas) was used for different institutions, so that the relative contribution of each form (area) to the global score depended on the type of institution being examined: Teaching Hospital, Scientific Research and Care Institute, General Hospital, Classified Hospital, Local Health Unit (LHU) Hospital.

Weights have been given using a Delphi approach. A panel of clinicians (medical doctors and surgeons) were asked to indicate the weights they considered most appropriate for the different healthcare organizations: the relative significance of the form areas was obtained for each type of organization, based on their case-mix index.

In order to facilitate the application of the methodology, a series of practical indications was also introduced and collected into an "interviewer handbook".

The internal consistency of the tool was measured by Chronbach's alpha.

### Software development

A software application has been developed for OPTIGOV based on a template from Microsoft Office products, to allow an easy interface with them. It has been structured into two main modules:

- the first one, based on a series of MS Excel sheets, for data interface and score processing;

- the second one, based on MS Access, carrying out data filling and processing.

Accordingly, the application allows three levels of interaction: interface, score processing and data processing (see below).

## Results

### The OPTIGOV methodology

The OPTIGOV methodology, aimed at the review of the degree of implementation of CG within a healthcare organization, is based on:

a) interviews, supported by a questionnaire, the Clinical Governance Scorecard (CGS);

b) review of hospital documentation;

c) data collection and analysis;

d) elaboration and development of operational plans for improvement, according to the priorities identified for the particular institution.

The OPTIGOV methodology consists of the following steps:

- analysis of CG structural and functional pre-requisites and areas;

- evaluation of these elements by a global score (with a maximum value = 100) derived from weighing of partial scores (subareas);

- identification of strengths and weaknesses of the organization;

- provision of suggestions and indications to the Hospital management, in order to trigger tangible improvement actions;

- creation of an up-to-date database with the results of the analysis;

- monitoring of health care services quality improvement.

### Questionnaire

The questionnaire consists of 13 analysis forms: the first 4 (indicated as A, B, C and D) refer to the essential structural and functional prerequisites for efficient adoption of the tools of CG, the other 9 (numbered as 1, 2, 3, 4, 5, 6, 7, 8 and 9) investigate the single CG areas and are aimed at the evaluation of the effective application level of each tool. CG tools are represented by all those instruments that ensure the pursuit of quality in health care (e.g. use of guidelines, clinical pathways and procedures, risk mapping, incident reporting systems, quality standards). OPTIGOV investigates the existence of CG tools within a health care organization through specific questions and evaluates their utilization or effectiveness level through a review process of hospital documentation. Each area is explored by a form with a variable number of questions and "closed" single or multiple answers; the total number of questions is 179 (Table 1). The OPTIGOV methodology allows a score to be assigned to each area of analysis, healthcare department and unit - the department being an organizational element defined by the Italian Ministry of Health as "an integrated organization of homogeneous, similar or complementary units [...] which all pursue common health outcomes [27]. The single scores can be gathered for the organization as a whole, thereby allowing for a global evaluation (see above).

**Table 1:** OPTIGOV analysis areas; first 3 questions of each form, including an example of weighted scores

ANALYSIS AREAS	Items	First 3 items of each form				
**CG structural and functional prerequisites:**						

A. Resources and Services Management	17	Do you have a planning and control system in place?	Yes, completely	Yes, partially	No	
		
		If so, does the attribution of objectives and resources to the single units occur by means of a formalised contractual process?	Yes	No		
		
		Is the planning process supported by a computerised procedure?	Yes	No		

B. Learning Culture	9	Have you defined a training plan?	Yes	No		
		
		If so, have health professionals been involved in the detection of training needs?	Yes, completely	Yes, partially	No	
		
		If so, are the training programs specifically targeted to the different professionals and responsibility levels?	Yes, completely	Yes, partially	No	

C. Research and Development	5	Are there units performing scientific research in the health care organization?	Yes	No		
		
		If so, are the research results shared with the other health care units?	Yes, always	Yes, sometimes	No, never	
		
		If so, are the research results used to modify health care protocols?	Yes, always	Yes, sometimes	No, never	

D. Information Technology	6	Is there a computerised system for management of clinical records?	Yes, completely	Yes, partially	No	
		
		Are legal and scientific references reported on the clinical records?	Yes, always	Yes, in most of the cases	No	
		
		Is there a computerised system for the outpatient clinical record management?	Yes, completely	Yes, partially	No	

**CG Areas**:						

1. Evidence-Based Medicine	21	In the process of taking decisions on patients, do doctors integrate their clinical experience with the best available scientific evidence?	Yes, always	Yes, sometimes	Yes, rarely	No, never
		
		If so, through which tool is this integration realised?	Exclusively by referring to databases (e.g. Cochrane Library)	Mainly by referring to databases (e.g. Cochrane Library)	Mainly by referring to scientific literature on paper) = 1,3888	Exclusively by referring to scientific literature on paper)
		
		If the source of information is computerised, where does the consultation occur?	In a handheld pc, at the bedside, = 4,1666	In a computer located within the unit = 2,0833	In a computer located within the department = 1,3888	In a computer located somewhere else within the hospital = 1,0416

2. Accountability	**9**	**A. Are medical activities accountable? **[*A question *= *4 Cs*]	**Yes, always = 19,0476**	**Yes, sometimes = 9,5238**	**No, never = 0**	
		
		**B. If so, is there a formalised accountability procedure? **[*B question = 2 Cs*]	**Yes = 9,5238**	**No = 0**		
		
		**C. If so, on what kind of support? **[*C question = base*]	**Both = 4,7619**	**Computerised = 2,3809**	**Paper = 2,3809**	

3. Clinical Audit	22	Is clinical audit performed within the units?	Yes, always	Yes, sometimes = 3,9214	No, never = 0	
		
		If so, how frequently is it performed ?	Weekly	Fortnightly	Monthly	Other
		
		Is there a formal documentation of the audits?	Yes	No		

4. Measurement of Clinical Performances	13	Is there an organizational structure specifically devoted to clinical performance measurement within the organization?	Yes	No		
		
		Are there professionals specifically devoted to clinical performance measurement within the organization?	Yes	No		
		
		If so, what kind of skills have they?	Both	Health care expertise	Management expertise	

5. Appraisal and Improvement of Clinical Activities	10	Do you use the measurement results in order to improve health care activity?	Yes, always	Yes, sometimes	No, never	
		
		Do you involve external high-level professionals in performance appraisal?	Yes, always	Yes, sometimes	No, never	
		
		Does the appraisal involve only the heads of the units or even doctors individually?	Even doctors individually	Only the heads of the units		

6. Health Technology Assessment	21	Is there a structure specifically devoted to Health Technology Assessment within the organization?	Yes	No		
		
		Are there professionals specifically devoted to Health Technology Assessment within the organization?	Yes	No		
		
		If so, what kind of skills have they? (multiple choice possible)	Management expertise	Health care expertise	Technical expertise	

7. Quality Systems	14	Do you have a quality system?	Yes	No		
		
		If so, is it certified by an accreditation and certification organization (certifying authority)?	Yes	No		
		
		If so, is the accreditation and certification organization specialised in certifying health care organizations?	Yes	No		

8. Risk Management	15	Have you performed a mapping of the existing risks in the units?	Yes	No		
		
		If so, has the risk mapping been communicated to all health professionals working within the unit?	Yes	No		
		
		Do you have an incident reporting system?	Yes	No		

9. Information, Citizen/Patient Involvement	17	Do you inform the patient of the clinical, diagnostic, therapeutic procedures he will undergo?	Yes, always	Yes, sometimes	No, never	
		
		If so, is the information supported by any documentation on paper (leaflets, brochures, forms) to be handed to the patient?	Yes	No		
		
		Are there case managers within the units?	Yes	No		

**TOTAL 13**	**179**					

Internal consistency, measured by Chronbach's alpha, showed good results for the vast majority of the areas (Table 2). The different weights given to the forms (areas) depending on the type of institution being examined are shown in Table 3.

**Table 2:** Results of the internal consistency test of the questionnaire areas

Area	Cronbach's alpha
Resources and Services Management	0.735

Learning culture	0.811

Research and Development	0.762

Information Technology	0.691

Evidence Based Medicine	0.856

Accountability	0.714

Clinical audit	0.723

Clinical performances measurement	0.845

Appraisal and improvement of clinical activities	0.622

Health Technology Assessment	0.610

Quality systems	0.778

Risk Management	0.899

Information, citizen's/patient's involvement	0.767

**Table 3:** Relative contribution of each form (area) to the global score in different types of institutions

	Teaching Hospital	%	Scientific Research and Care Institute	%	General Hospital	%	Classified Hospital	%	LHU Hospital	%
A - Resources and services management	10	7.69	10	7.69	8	6.78	7	6.25	5	5

B - Learning culture	10	7.69	10	7.69	8	6.78	7	6.25	5	5

C - Research and development	10	7.69	10	7.69	8	6.78	7	6.25	5	5

D - Information technology	10	7.69	10	7.69	8	6.78	7	6.25	5	5

1 - Evidence Based Medicine	10	7.69	10	7.69	10	8.47	10	8.93	10	10

2 - Accountability	10	7.69	10	7.69	10	8.47	10	8.93	10	10

3 - Clinical audit	10	7.69	10	7.69	10	8.47	10	8.93	10	10

4 - Clinical performances measurement	10	7.69	10	7.69	10	8.47	10	8.93	10	10

5 - Appraisal and improvement of clinical activities	10	7.69	10	7.69	10	8.47	10	8.93	10	10

6 - Health Technology Assessment	10	7.69	10	7.69	8	6.78	7	6.25	5	5

7 - Quality systems	10	7.69	10	7.69	10	8.47	10	8.93	10	10

8 - Risk Management	10	7.69	10	7.69	8	6.78	7	6.25	5	5

9 - Information, citizen's/patient's involvement	10	7.69	10	7.69	10	8.47	10	8.93	10	10

**Teaching Hospital: **organization structured as a company, of national or super national level, dedicated to healthcare services associated with didactics and research.

**Scientific Research and Care Institute: **organization structured as a company, of national or super national level, dedicated to high specialty healthcare services associated with biomedical research.

**General Hospital: **organization structured as a company of regional or national level for healthcare services.

**Classified Hospital: **religious hospital considered equal to the General Hospital within the National Healthcare System.

**LHU (Hospital under the jurisdiction of the Local Health Unit): **hospital with a medium-to-low level of healthcare services complexity (e.g. prevention, healthcare to elderly people, etc.) which are directly managed and funded by Local Health Units.

The examiners are members of a Project Team, composed of public health professionals and management analysts belonging to:

- the Clinical Governance Unit of the Institute of Hygiene of the Catholic University of the Sacred Heart, Rome, Italy;

- the Public Health and Microbiology Department, University of Turin, Italy.

They are therefore drawn from outside the organization under review, to ensure the objectivity of the diagnostic review. Project Team members are carefully trained in the knowledge and application of the methodology by the multidisciplinary team who developed the methodology. Questionnaire is completed face to face by the expert compilers of the Project Team, who record answers provided by interviewees.

### Software application

The software application allows the expert compilers who perform audits to collate all of the required data, calculate the scores, make comparisons intra- and inter-healthcare organizations and develop benchmarks.

The application consists of three levels:

1. the first level is the interface used by the expert to register the answers electronically;

2. the second level is the score processing, which provides scores referring to areas, departments, units and hospital;

3. the third level is the data processing, useful in carrying out comparisons, formulate statistics and indicators and create graphics.

The interfaces provided are identical to the paper questionnaire, so that the compiler can decide to fill in the questionnaire on his laptop or do it afterwards.

The questions are structured in a hierarchical order: there are basic questions ("mother questions") that trigger other questions ("child questions"). The hierarchy influences the scoring system: the "mother question" has a heavier weight than the "child question" (see above).

The database is structured in three hierarchical levels: level 1 includes all the separate questions, without grouping or aggregation, level 2 includes the questions recorded by area and by unit - each area can refer to many units - and level 3 includes the questions recorded by department and whole health care organisation.

### Interviews, Interviewees and data sources

**Figure 1:**
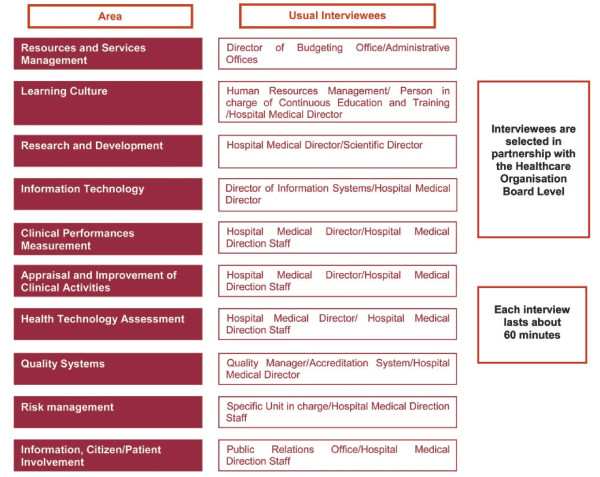
**Board level Interviews**.

**Figure 2:**
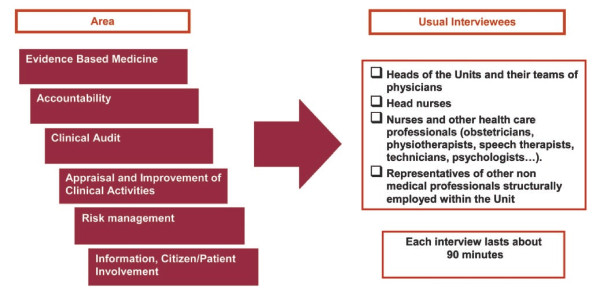
**Unit level Interviews**.

There are two different interviews levels and models: Board and Unit. The Board is represented by the top management of the health care organization, the Unit is represented by each single ward. Board level interviews require 10 forms to be administered: A, B, C, D, 4, 5, 6, 7, 8, 9; Unit level interviews require 6 forms to be administered: 1, 2, 3, 5, 8, 9. Interviewees, who are represented by health professionals operating at all levels within the organization, are selected by the Project Team in partnership with the healthcare organization's Board, which is aware of the specific roles and levels of responsibility within the organization and can therefore indicate the most appropriate interlocutors to report on specific areas. Figures 1 and 2 show the usual interviewees selected for the Board and Unit interviews respectively. In order to review hospital documentation and collect data about its structural features (e.g. number of departments, units, beds), human resources (e.g. number of doctors, nurses, technicians) and activity indicators (e.g. value of production, number and average weight of hospitalizations, beds occupation rate, surgical cases %), a series of data sources must be consulted (Table 4).

**Table 4:** Essential data sources to be reviewed

Health Care Organisation Chart
Processes Maps

Procedures for risk classification and reduction

Procedures and Formal Documents (i.e. budgeting, meeting minutes, etc.)

Documents of Reform Projects

Periodic Education Plan

Clinical Care Pathways

Clinical Records

Operating Theatres Registries

Performance Measurement or Appraisal Tools and Procedures

Health Care Professionals Evaluation Forms

HTA Tools and Procedures

Procedures for Privacy Management

Brochures and Booklets for Patients

Customer Satisfaction Questionnaires

### Actions

OPTIGOV requires a specific sequence of actions to be taken. The Project Team is created (see above) which identifies areas of specific interest (high level management and management staff, administration and health departments and units), carries out the diagnostic review and the evaluation activity via hospital audits by acquiring a set of pre-established data, performing interviews and reviewing hospital documentation. Information gained through the interviews are then compared with the contents of the documentation and finally filled in the registration forms.

**Figure 3:**
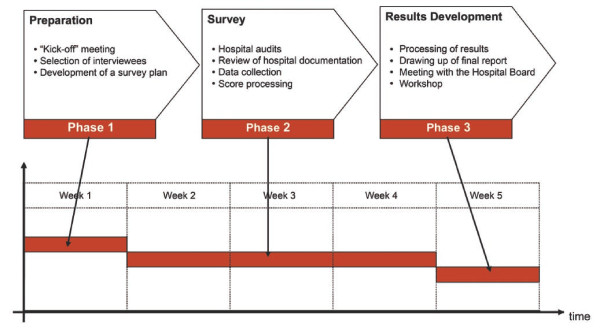
**Timeframe of the OPTIGOV methodology application**.

Data are then processed, and a set of evaluations and scores per area, department, unit and hospital is produced. The results of the review are summarized in a final report. The report should indicate strengths and weaknesses, criticalities and transferable best practices of the healthcare organization; provide suggestions and indications to the Management; describe an operational plan for change which specifies the possible future priorities and interventions for improvement. The timetable may include short, middle and long term changes, depending on the criticalities detected. After a meeting with the Board for a first evaluation of the results of the analysis, a final workshop is set up to share the conclusions with all the stakeholders. The timeframe of application of OPTIGOV is about 5 weeks (figure 3).

The next stage is represented by the implementation of the proposed changes in an operational plan: the analysed healthcare organizations undertake specific improvement actions, in order to increase the quality of services and processes. Both the Project Team and the Board of the healthcare organization are involved in the implementation process. The effective degree of improvement of the quality of services is monitored by further diagnostic reviews, the results of which are compared with the previous organization status.

## Discussion

It is widely accepted that the term CG describes an organizational accountability framework useful to improve clinical care, safeguard standards and work towards excellence [28]. The introduction of CG into the National Health Service can be seen as a fundamental shift in the regulatory relationship between the state and medical professionals [29], while at the organizational level it can be considered a process that involves a move towards "encoded knowledge" through the use of "soft bureaucracy" [30]. In this view, according to Iedema et al (2005), one can distinguish between moralizing and disciplinary devices, the latter useful for inspecting data generation and analysis, performance monitoring and management, accreditation, guidelines and protocol production and implementation, and the close integration of clinical and financial data [31].

The OPTIGOV methodology, described in this article, can be seen in the perspective of the above mentioned disciplinary devices. In particular, it may appear to be similar in nature to hospital accreditation protocols [32-35] and a degree of overlap does in fact exist. Nevertheless, the distinctive perspective of OPTIGOV is focused on the existence and the level of implementation of CG tools. Unlike accreditation agencies, OPTIGOV does not issue any certifications, but is solely aimed at implementing CG and improving the quality of health care. Therefore traditional accreditation agencies and OPTIGOV reflect a different culture towards quality improvement in those who choose them and could have a different impact on the cultural change of health professionals operating at all levels within the organization.

Furthermore, although attempts to address the question of organic and flexible evaluation of the implementation of CG have already been made, the evaluation systems they have produced are either experimental ones, with a broadly qualitative approach [24] or else they suffer from a limited scope, as they were designed for very specific sectors or one-off studies [25,26].

Surveys on the effectiveness of good practices in medicine have revealed that the single most important problem in their application is lack of dedicated time and resources as well as low motivation on the management side [36-39]. These observations suggest that, for good practices to be effective in a single institution, they must not only be known to health professionals, but they must represent a permanent part of work routines; and that it is reasonable to ascertain, as OPTIGOV does, that they are a well-defined component of normal behaviours and a priority shared by all members of the medical equipe - which may not be the case, even when physicians maintain they know the essentials of those practices.

An important aspect addressed since the early stages of the development of OPTIGOV was the choice of the subjects to be held responsible for each area and interviewed. Over the last decade, debate over the accountability in implementing CG has suggested that responsibility is shared by managers and physicians alike, though to different degrees [11,41,42]. This observation has been taken into account by the authors in establishing how to determine who should be interviewed about what.

The choice of the areas to be explored and the subareas to be considered as their components was based on the earlier definitions of CG, but subsequent discussion on the real contents of good practices as well as questions that have been raised by health care reforms were taken into account. In particular, consideration of the growing trend of transferring scientific research into more and more levels of health care and the subsequent need to evaluate research skills [43] supported the inclusion of the area "Research & Development" and the careful weighing of its contribution to the final score. The controversies over the perceived importance and the evolution of the practice of Clinical Audit [44,45] were also considered when developing the correspondent set of questions and scores. Especially in the field of error and risk management, all of the aspects that were signalled as relevant in the literature were included [46,47].

OPTIGOV is currently being put to the test in the setting of several projects, at the moment restricted to Italy, involving a number of health care institutions of different types [48]. The next step in its development will thus be the evaluation of the results of its application. The opinions of health administrators will be collected, so as to check for any weaknesses in its completeness, effectiveness, ease of applicability and flexibility. In particular, the ability of the results of an OPTIGOV diagnostic review to establish priorities, provide basis for tailored interventions and influence subsequent clinical and management decisions has already been partially assessed after the first testing of OPTIGOV [48]. The latter led in fact to the triggering and implementation of a series of improvement actions (e.g. activation of training programmes on evidence-based medicine and clinical audits, and definition and dissemination of risk management procedures), some of which are still in progress.

A long-term analysis of the effect of an OPTIGOV diagnostic review on the level of implementation of CG in the structures involved will also serve as an indirect evaluation of the improvement actions that have been introduced following the reviews themselves. The effectiveness of these actions will be tested through a comparison of data before and after the interventions prompted by OPTIGOV.

OPTIGOV also offers the opportunity to make intra- and inter-organizations comparisons, by showing differences in the level of adoption and spreading of adequate CG tools.

The development of the methodology could have been affected by some limitations. First, the selection of the areas to be considered and the questions they include could be questionable; however, we selected the areas on the basis of the definition of CG and a review of the scientific literature on this issue. Moreover, the first results of OPTIGOV [48] lead to hypothesize the reliability and reproducibility of the methodology.

Another critical point could be represented by the way data are collected in OPTIGOV and the risk of information bias. The main element which controls information bias is the double origin of data: face-to-face interviews of single professionals indicated by the organization's Board are the first source; then, the relevant official documentation of the organization is consulted, and it is compared to the information provided by the interviewees so as to correct it if contradictions are detected.

## Conclusions

The OPTIGOV methodology aims to assess the CG implementation level within health care organizations. The results of the ongoing OPTIGOV implementation projects will allow to verify the following aspects of the methodology, based on scientific literature and on the observations of health administrators (see above):

- its completeness and its coverage of the main CG best practices so far identified;

- its accuracy in the choice of the indicators used to demonstrate the level of application of each practice;

- its applicability and capability of covering different types of health care institutions.

OPTIGOV has the potential to produce a realistic representation of the organization status, to pinpoint both criticisms and transferable best practices. Thus it provides concrete plans for organizational change that increase the likelihood of improvements in the quality and excellence of health care.

## Competing interests

The authors declare that they have no competing interests.

## Authors' contributions

GLT, RS, WR reviewed scientific literature, chose the analysis areas and developed the questionnaire; MLS, SC, PN, LV drafted the manuscript; AC developed the scoring system and the software. All the authors read and approved the manuscript.

## Pre-publication history

The pre-publication history for this paper can be accessed here:

http://www.biomedcentral.com/1472-6963/10/174/prepub
